# Modified home range kernel density estimators that take environmental interactions into account

**DOI:** 10.1186/s40462-019-0161-9

**Published:** 2019-05-21

**Authors:** Guillaume Péron

**Affiliations:** Univ Lyon, Université Lyon 1, CNRS, Laboratoire de Biométrie et Biologie Evolutive UMR5558, F-69622 Villeurbanne, France

**Keywords:** Movement ecology, AKDE, Temporal autocorrelation, Step selection function, Resource selection, Point process pattern, Semiparametric

## Abstract

**Background:**

Kernel density estimation (KDE) is a major tool in the movement ecologist toolbox that is used to delineate where geo-tracked animals spend their time. Because KDE bandwidth optimizers are sensitive to temporal autocorrelation, statistically-robust alternatives have been advocated, first, data-thinning procedures, and more recently, autocorrelated kernel density estimation (AKDE). These yield asymptotically consistent, but very smoothed distributions, which may feature biologically unrealistic aspects such as spilling beyond impassable borders.

**Method:**

I introduce a semi-parametric variant of AKDE designed to extrapolate more realistic home range shapes by incorporating movement mechanisms into the bandwidth optimizer and into the base kernels. I implement a first approximative version based on the step selection framework. This method allows accommodating land cover selection, permeability of linear features, and attraction for select landscape features when delineating home ranges.

**Results:**

In a plains zebra (*Equus quagga*), the reluctance to cross a railway, the avoidance of dense woodland, and the preference for grassland when foraging created significant differences between the estimated home range contours by the new and by previous methods.

**Conclusion:**

There is a tradeoff to find between fully parametric density estimators, which can be very realistic but need to be provided with a good model and adequate environmental data, and non-parametric density estimators, which are more widely applicable and asymptotically consistent, but whose details are bandwidth-limited. The proposed semi-parametric approach attempts to strike this balance, but I outline a few areas of future improvement. I expect the approach to find its use in studies that compare extrapolated resource availability and interpolated resource use, in order to discover the movement mechanisms that we need to improve the extrapolations.

**Electronic supplementary material:**

The online version of this article (10.1186/s40462-019-0161-9) contains supplementary material, which is available to authorized users.

## Introduction

Many researchers use kernel density estimators (KDE) to extrapolate where a geo-tracked animal spends its time, often using the 95% extrapolated isopleth as the home range contour [1–3]. KDE work by approximating the stationary utilization distribution *p*(***r***) of the animal, i.e., its time budget with respect to location ***r***, with a sum of “kernels”, i.e., unimodal distributions *κ* centered around each recorded location {***r***_***i***_} [4, 5].

1 $$ \widehat{p}\left(r,\left\{{\boldsymbol{r}}_{\boldsymbol{i}}\right\},{\boldsymbol{\upsigma}}_{\boldsymbol{B}}\right)=\frac{1}{N}\sum \limits_{i=1}^N\kappa \left(\boldsymbol{r},{\boldsymbol{r}}_{\boldsymbol{i}},{\boldsymbol{\upsigma}}_{\boldsymbol{B}}\right) $$

The parameter **σ**_***B***_, termed the bandwidth, controls the spread of each kernel around each recorded location, and therefore ultimately the degree of smoothing of the resulting distribution [4, 6, 7]. A small bandwidth yields a distribution with numerous peaks around each cluster of recorded locations; a large bandwidth smooths out these peaks and yields a more spread-out distribution [2, 5]. The choice of an appropriate bandwidth is therefore critical, and indeed usually trumps the influence of the actual shape of the kernels, i.e., the analytical form of function *κ* [4]. At least three categories of optimal bandwidth selection routines have been developed to inform and automatize this decision [8], but, because of temporal autocorrelation in movement data [9–11], only one, the reference function approximation, is recommended for animal tracking applications [12]. In the reference function approximation approach, one optimizes the bandwidth by minimizing an approximated mean integrated square error criterion (MISE).

2 $$ \mathrm{MISE}\left({\boldsymbol{\sigma}}_{\boldsymbol{B}}\right)=\left\langle {\int}_{\Omega}{\left|{p}_{REF}\left(\boldsymbol{r}\right)-\widehat{p}\left(\boldsymbol{r},\left\{{\boldsymbol{r}}_{\boldsymbol{i}}\right\},{\boldsymbol{\upsigma}}_{\boldsymbol{B}}\right)\right|}^2\ d\boldsymbol{r}\right\rangle $$

*p*_*REF*_(***r***) is the reference function, usually chosen for its mathematical properties. In a non-approximate MISE, there should be a *p*(***r***) term instead of *p*_*REF*_(***r***), but, since *p*(***r***) is the unknown we want to estimate, we need to replace it with the reference function. Ω represents the domain of possible locations, the ∫ ∙ *d****r*** notation denotes integration over one realization of the stochastic movement process in space, and the 〈∙〉 notation denotes integration over all realizations of the movement process.

If Eq. 2 did not account for the temporal autocorrelation structure of the movement process, it would introduce a bias in the bandwidth estimate, that can severely impedes comparative inference [7, 9, 11–15]. For example, the optimizer would converge towards a zero bandwidth when the sampling resolution would increase or the amount of temporal autocorrelation in the animal movement would increase, yielding increasingly smaller home ranges [12]. The amount of bias depends on *both* the sampling resolution and the movement rates of the animal. There are two known ways to deal with temporal autocorrelation in Eq. 2. First, one may subsample the data so that successive records are approximatively independent [9, 16]. The recommended best practice is to keep one record every 3*τ* where *τ* is the autocorrelation time (the rate at which the proximity between two records declines with the time lag between them). I hereafter refer to this as “robust KDE” (KDEr). The other way to deal with temporal autocorrelation is to keep all the data in, but specify a temporal autocorrelation model in the kernels that make up $\widehat{p}$ and in the reference function, yielding a different MISE that is minimized by a different value of the bandwidth [12]. This approach is termed “autocorrelated kernel density estimation” (AKDE). In both cases, the approximation of *p*(***r***) by *p*_*REF*_(***r***) still leads to a “reference function approximation bias” [17]. This bias is usually positive. It can be corrected a posteriori [17], in which case I use the recommended notation “c”, e.g., AKDEc.

Both these robustizing protocols increase the bandwidth compared to standard or naive KDE. The KDEr option also requires the user to discard potentially massive amounts of data. As a consequence, the resulting distribution, although statistically robust and asymptotically consistent, may look oversmoothed and biologically irrelevant [16, 18]. For example, the 95% isopleth might intersect impassable barriers such as coastlines [19]. The issue of boundaries is indeed recurrent in KDE applications, including in other fields than animal tracking data analysis (review in [20, 21]). For home range estimation, the most common response is to clip the distributions at known barriers [19]. This intuitive practice is equivalent to introducing a dose of mechanism in the otherwise fully nonparametric KDE methodology. By specifying where to clip and how to redistribute the weight, we in effect inform the model that the barrier was unpassable. However, instead of providing that information in an ad hoc way at the very end of the process, we could uptake it from the start. The MISE would then become less sensitive to an increase in bandwidth that would otherwise have caused the kernel distributions to spill beyond the barrier, yielding a different optimal bandwidth. We would also incorporate the barrier into the reference function, making it more realistic, and thereby suppressing the reference function approximation bias. Lastly, we would apply the effect of the barrier to each kernel, yielding an estimated utilization distribution that is truncated at the barrier by construct. More generally, in addition to barriers, we can incorporate in this way any movement mechanism that can be formalized using a step selection function [22, 23], e.g., land cover selection [22] and permeability of linear features like roads [24].

## Material and methods

### Step 1: fitting a mechanistic movement model

At time *t*, the position ***r***_*t*_ of the animal was assumed to be drawn from a step selection kernel *g*_*u*_ made of the product of an availability function *g*_*a*_, conditioned on the movement path prior to *t*, ***R***_*t* − 1_, and especially the last known position, ***r***_*t* − 1_, and of a weight function *W*, both defined over the movement domain Ω [22, 23, 25].

3 $$ {g}_u\left({\boldsymbol{r}}_t|{\boldsymbol{R}}_{t-1}\right)={K_t}^{-1}W\left({\boldsymbol{r}}_t|{\boldsymbol{R}}_{t-1},t\right){g}_a\left({\boldsymbol{r}}_t|{\boldsymbol{R}}_{t-1}\right) $$

*K*_*t*_ is a scaling constant so that *g*_*u*_ sums to one.

The availability function *g*_*a*_ was modelled using the Ornstein-Uhlenbeck process (OU), a continuous-time stochastic movement model that represents home range behavior as a tendency to revert back to a mean location following random deviations from that mean [26]. The weight function *W* described environmental interactions: selection of some land covers over others, attraction or repulsion towards fixed landscape features such as human settlements, and barrier permeability, i.e., the rate at which animals avoid crossing linear features such as roads and rivers [25]. Following established practice [22, 27, 28], the analytical form of the weight function *W* was:

4 $$ \log\ W\left({\boldsymbol{r}}_t|{\boldsymbol{R}}_{t-1},t\right)=\boldsymbol{x}{\left({\boldsymbol{r}}_t\right)}^{\mathrm{T}}\cdot \boldsymbol{\alpha} +\boldsymbol{\delta} {\left({\boldsymbol{r}}_t|{\boldsymbol{r}}_{t-1}\right)}^{\mathrm{T}}\cdot \boldsymbol{\lambda} $$

***x***(***r***_*t*_) describes the environment at location ***r***_*t*_. The *k*^th^ elements of ***x***(***r***_*t*_) (*k* = 1, … *K*_1_) contains a 0 or 1 coding for the presence of land cover type *k* at location ***r***_*t*_. The next *K*_2_ elements contain the distances from ***r***_*t*_ to fixed landscape features (or the value of continuous environmental covariates such as climate or vegetation density). ***δ***(***r***_*t*_| ***r***_*t* − 1_) is a vector containing a 1 in the *l*^h^ cell if a barrier of type *l* is crossed when going straight from ***r***_*t* − 1_ to ***r***_*t*_. Note that this straight-line permeability model is valid only for small steps that make longer detours extremely unlikely.

The model parameters are included in vectors ***α*,** the selection coefficients, and ***λ***, the permeability coefficients. The *k*th element of ***α*** (1 < *k* ≤ *K*_1_) quantifies how much land cover type *k* is preferred over land cover type 1. The *l*th element of ***λ*** quantifies the reluctance to cross linear features of type *l*, zero meaning that the linear feature has no effect on movement. Both ***α*** and ***λ*** were considered constant through time and space in the present application.

For the sake of simplicity, and because my focus here was on the post-fitting treatment of ***α*** and ***λ*** estimates, rather than on the estimation itself, I used a relatively fast but approximate procedure to estimate the parameters in *W.* Following Johnson, Hooten & Kuhn [29], I reformulated the movement model as a space-time point process [30, 31]. This meant that the availability function *g*_*a*_ was approximated by a Brownian availability window at each time step. However, in the post-fitting treatment, I used the Ornstein-Uhlenbeck model for *g*_*a*_ as announced above. This means that different contradictory models were in practice used to estimate the weight function and the availability function. The detail of the space-time point process implementation is provided in Additional file 2.

### Step 2: bandwidth optimization

To incorporate environmental interactions, I replaced the kernels of Eq. 1 by weighed multivariate Gaussian distributions [20, 21].

5 $$ \widehat{p}\left(\boldsymbol{r}\right)=\frac{1}{N}\sum \limits_{i=1}^N{K_i^B}^{-1}W\left(\boldsymbol{r}|{\boldsymbol{R}}_i\right)\varphi \left(\boldsymbol{r},{\boldsymbol{r}}_{\boldsymbol{i}},{\boldsymbol{\upsigma}}_{\boldsymbol{B}}\right) $$

*φ*(***r***, ***r***_***i***_, **σ**_***B***_) denotes the multivariate Gaussian distribution of mean ***r***_***i***_ and variance-covariance matrix **σ**_***B***_, and ${K}_i^B$ is a scaling constant so that each kernel sums to one (more details in Additional file 1). Following Fleming et al. [12], I simplified Eq. 5 using ***σ***_*B*_ = *σ*_*B*_ ∙ ***σ***_0_, where ***σ***_0_ is the variance-covariance matrix of the availability function. This means that the direction of the smoothing was driven by the anisotropy of the movement process.

Importantly, these kernels feature a simplistic permeation model that imply a straight-line move from ***r*** to ***r***_***i***_ (Eq. 4**)**. In the zebra case study below, the violation of that straight-line assumption was almost without consequence, because the *σ*_*B*_ value was moderate and the linear feature exhibited no convolution at all, meaning that even if the path from ***r*** to ***r***_***i***_ was not straight, the chance to cross the linear feature was similar to that of a straight move. However in other applications, researchers may need to consider alternative jackknifing methods, e.g., diffusive permeation kernels [32]. In addition, with Eq. 5 we assume a regular redistribution of the discounted weight across the whole availability domain. In applications where the animals remain near the linear features when they encounter them [19], alternative redistribution rules may be warranted, e.g., reflected kernels [20].

Next, for the reference function, I also used weighted multivariate Gaussian distributions, summed over recorded locations to represent the expected equilibrium distribution of the movement process.

6 $$ {p}_{REF}\left(\boldsymbol{r}\right)=\frac{1}{N}\sum \limits_{i=1}^N{K_i^0}^{-1}W\left(\boldsymbol{r}|{\boldsymbol{R}}_{\boldsymbol{i}}\right)\varphi \left(\boldsymbol{r},{\boldsymbol{\mu}}_{\mathbf{0}},{\boldsymbol{\upsigma}}_{\mathbf{0}}\right) $$

${K}_i^0$ is a scaling constant so that each element of the reference function sums to one.

Like Eq. 5, Eq. 6 features a simplistic permeation model that assumes a straight line move from ***μ***_**0**_ to ***r***. However, now that simplistic permeation model is applied to the whole home range. Any movement bottleneck, e.g., a constrained corridor between two sections of the home range, would be overly discounted in the resulting reference function. In the zebra case study, we did not have to deal with any such feature. However, in other applications, users may have to consider alternative formulations. Individual-based simulations (IBS; see “Zebra case study” below) could in this case prove particularly useful to generate a “pilot estimate” to use instead of Eq. 6. In addition to offering a straightforward way to incorporate above-mentioned movement bottlenecks into the reference function, IBS can be set up so that the simulation step length is short enough that the straight-line permeability model remains realistic at all stages of the simulations. However, this option is not implemented yet, and probably warrants further investigation pertaining to sensitivity to simulation parameters (simulated duration, step lengths, etc.).

When using Eq. 5, 6, there is an analytical form for the MISE, derived in Additional file 1. These details expand on the proof of Fleming et al. [12] demonstrating how temporal autocorrelation is incorporated into the KDE framework. The resulting MISE optimization algorithm is however prohibitively time-consuming. Thus, pending algorithmic improvement, the new approach remains mostly theoretical and exploratory. I however developed a faster-running simplified version in the following section.

#### Simplified version

Because the MISE optimization was so prohibitively time-consuming, I simplified the protocol by by-passing Step 2 entirely, or more precisely, replacing Step 2 with the corresponding step of the AKDE analytical protocol [33]. In other words, the bandwidth is optimized while taking temporal autocorrelation into account, but without taking environmental interactions into account. The weights from Step 1 are then only applied when eventually computing the distribution (Step 3). I propose the notation E-AKDE for the full version where environmental interactions are incorporated in Step 2, and SE-AKDE for the simplified version where environmental interactions are not incorporated at Step 2. Importantly, because of the assumption from the straight-line permeability model, E-AKDE is not compulsorily always more reliable than SE-AKDE.

### Step 3: computing the kernel density and correcting for the remaining reference function approximation bias

From the estimated *σ*_*B*_, I computed isopleths of $\widehat{p}\left(\boldsymbol{r}\right)$ using Eq. 5. I then applied the reference function bias correction routine of Fleming and Calabrese [17] to the isopleths, but only when implementing SE-AKDE, hence the SE-AKDEc notation hereafter. When implementing E-AKDE, I considered that by changing the reference function (Eq. 6), I got rid of the reference function approximation bias. This is certainly a strong assumption that was however supported by the empirical results.

### Zebra case study and comparison with alternatives to KDE

I analyzed data collected from a plains zebra (*Equus quagga*) in and near Hwange national park, Zimbabwe (26.861E, − 18.624 N). The study individual (individual local identifier: Ganda) was monitored between Jan 2011 and Sept 2012 with a collar-mounted GPS that recorded one location every hour [34]. I rescaled the recent Hwange vegetation map [35] at a 150 m resolution and pooled vegetation classes into 4 categories to reduce computing time for this illustration case. Other landscape features known to influence zebra space use included water holes, a railway with adjacent road that marks the northern border of the park, and a town. These were all included in the step selection model (Eq. 3).

I compared four variants of the KDE methodology: a naïve reference function-based bandwidth optimizer that did not account for temporal autocorrelation (KDE), the robustized approach where the data were subsampled before analysis (KDEr), the AKDE approach where the autocorrelation structure was incorporated in the reference function (AKDEc), and finally the new methods (E-AKDE and SE-AKDEc). For KDE and KDEr I used the kde2d function in R-package MASS. The position autocorrelation time *τ* required to subsample the data for KDEr was estimated from the ctmm.fit routine in R-package ctmm [33]. For AKDEc, I used the akde function in ctmm within the recommended analytical protocol [33]. For E-AKDE, the algorithm and mathematical justification are described (with words) in Additional file 1. For SE-AKDEc, I used the AKDE bandwidth but then applied the environmental interaction weights when computing the distribution.

I also implemented three non-KDE methodologies. First, I used the asymptotic distribution of the fitted Ornstein-Uhlenbeck model to draw the ellipses that most closely approximated the home range and core area. Second, I implemented the movement-based kernel density estimator (MKDE), in which the kernels are replaced by step selection functions, i.e., the *g*_*u*_(***r***_*i* + 1_| ***R***_*i*_) estimated at Step 1 [19, 36]. MKDE, like the Brownian bridge, computes the probability that a location was used *between records* [36]. The method focuses on *one* realization of the movement path and on the process uncertainty around the interpolated path between records [37]. By contrast, KDE extrapolators average the utilization distribution *across realizations* of the movement path. Lastly, I implemented an individual-based simulation procedure (IBS) [38, 39] to generate 1000 1-month-long tracks with one record per hour, each track starting from a randomly selected recorded location, and moving stochastically according to the model described in Eq. 1. This yielded a cloud of 720,000 simulated records, from which I computed the density of records per pixel, thereby obtaining a rasterized cumulative utilization distribution that quantified the time budget under the fitted mechanistic movement model.

For each estimator, I computed the home range area (95% isopleth) and the core area (50% isopleth), as well as the home range scale computed as the root mean square distance of the distribution to its centroid. I also computed the “amplitude” of the core area and of the home range as the longest distance between two points on the isopleth. These represent different ways to measure the home range. In particular, the home range area of the asymptotic OU distribution is proportional to the movement variance or home range scale.

## Results and discussion

### Comparison between KDE variants in the zebra case study

**Fig. 1:**
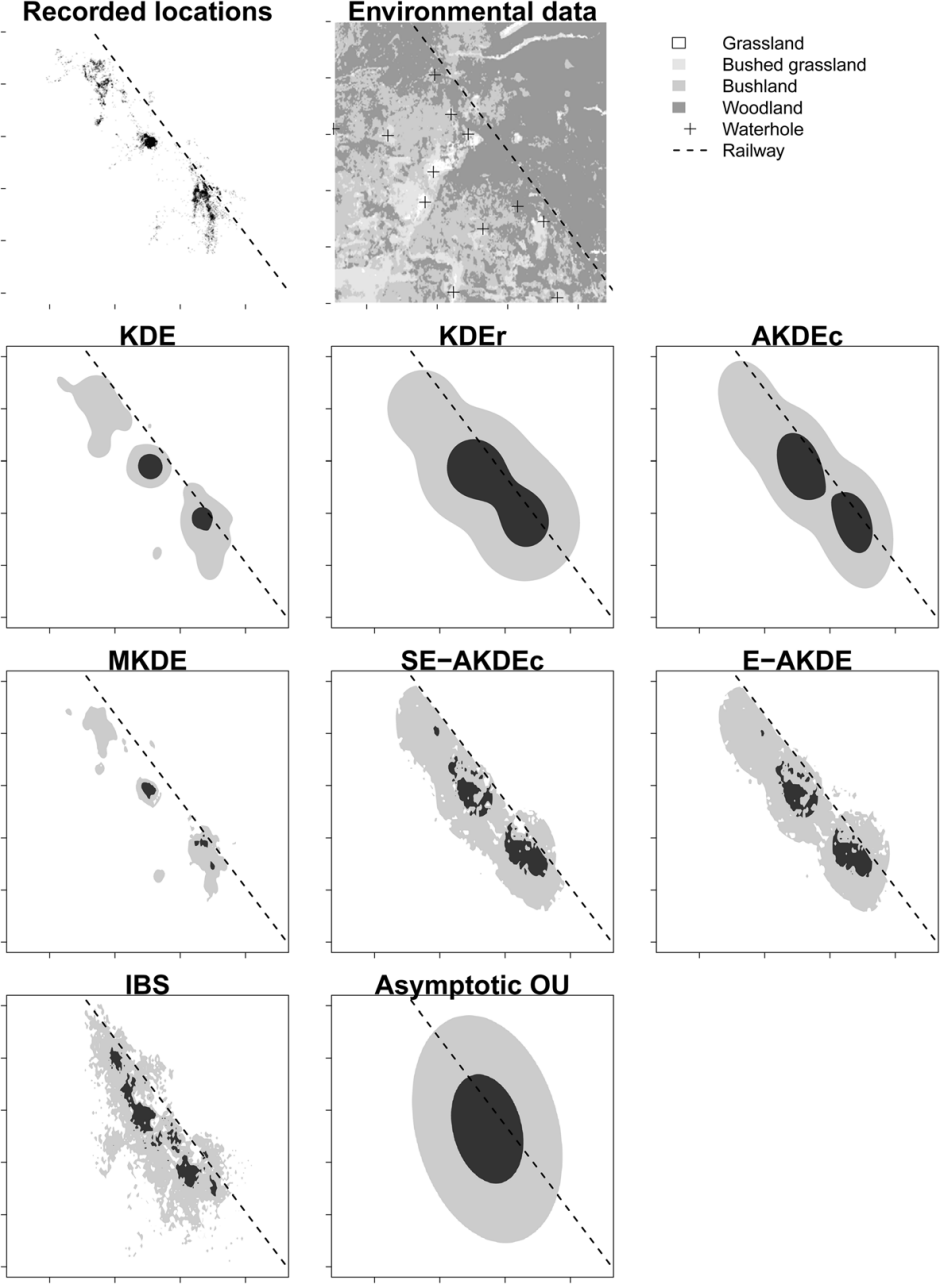
The 50% (dark grey) and 95% (light grey) isopleths of the utilization distribution of a plains zebra, as estimated by the 8 estimators in this study. The dashed diagonal line represents a railway with adjacent road that marks the border of the national park where the zebra was captured. KDE, KDEr and AKDEc represent three strategies to choose the bandwidth of the kernel density estimator. MKDE is a bridge-based interpolation of the movement path. SE-AKDEc and E-AKDE are the result of the new developments in this study, incorporating step-selection functions into the AKDE framework. IBS depicts a cloud of 720.000 simulated locations from a fitted step-selection model. The asymptotic OU distribution represents the spread of a simple Ornstein-Uhlenbeck advection-diffusion model fitted to the data

The standard KDE yielded the smallest home range area and amplitude among all KDE variants (Fig. 1 and Table 1). As reviewed in the introduction, this small estimated home range size is partly caused by an unwanted property of the standard KDE in the presence of temporal autocorrelation [7, 9, 11–15]. The KDEr version indeed yielded a much larger estimated home range. AKDEc yielded a smaller home range estimate than KDEr, with a notably smaller core area leading to a large estimated home range scale. Neither AKDEc nor KDEr provided any information about landcover selection or reluctance to cross the railway (Fig. 1), as expected by construction. The most visually compelling effect of using SE-AKDEc and E-AKDE was that the space to the east of the railway was weighted down compared to AKDEc and KDEr. In addition, the core area was markedly irregular in shape, reflecting the avoidance of the densest woodland cover type and the preference for pure grassland. The composition of the home range remained similar across all methodologies. In particular, while c.30% of the raw data was recorded in grassland, only 1–6% of the estimated home range was estimated to be grassland. This result stems from the scarcity and patchiness of grassland in the area, meaning that any extrapolation was bound to incorporate more non-grassland than grassland. In addition, bushland and woodland are sometimes actively selected by zebras, e.g., after a predation event [34], meaning that the fitted landcover selection model did not strictly discount these landcover types and that it might be interesting to fit a time-varying landcover selection model in this context. Lastly, the estimated E-AKDE bandwidth (0.20) was smaller than that of AKDE (0.31) yielding a smaller estimated home range from E-AKDE than SE-AKDEc. The decrease in bandwidth suggests that the methodology successfully took up the information that some of the movement variance was caused by resource selection, not stochastic diffusion. These empirical results overall suggest that the new reference function suppressed the reference function approximation bias.

**Table 1:** Home range area, home range scale, and home range composition for the same zebra study individual, using the various home range estimation methods. The home range scale is the root mean squared distance to the distribution centroid. The amplitude is computed as the longest distance between two points on a contour. IBS stands for individual-based simulation, other notation like in the main text

	Core area size (50% isopleth) [km^2^]	Home range size (95% isopleth) [km^2^]	Home range scale [km]	Core area amplitude (50% isopleth) [km]	Home range amplitude (95% isopleth) [km]	Proportion of grassland in core area (50% isopleth) [%]	Proportion of grassland in home range (95% isopleth) [%]	Proportion of woodland in core area (50% isopleth) [%]	Proportion of woodland in home range (95% isopleth) [%]
MKDE	4.3	46.9	14.1	13.8	29.1	24.3%	3.1%	21.6%	66.9%
KDE	12.9	117.3	14.0	12.7	31.1	6.1%	1.5%	68.2%	69.4%
KDEr	81.0	332.6	14.2	16.7	32.0	2.3%	1.6%	76.4%	64.3%
AKDEc	62.8	264.7	18.5	17.1	31.6	2.1%	1.3%	73.3%	66.8%
SE-AKDEc	38.5	229.5	14.0	22.1	32.6	12.3%	7.1%	10.9%	8.8%
E-AKDE	29.0	190.4	14.0	21.7	31.1	13.3%	6.3%	11.5%	9.8%
IBS	28.1	162.4	10.7	21.4	32.7	11.9%	10.0%	0.0%	2.0%
Asymp. OU	88.6	383.0	14.0	12.1	25.1	6.2%	7.3%	28.3%	24.0%

### Extrapolation/interpolation, parametric/non-parametric

As outlined by several authors previously, MKDE does not measure the same thing as KDE [33, 36]. MKDE implements an *interpolation*. The potential for confusion has led some authors to recommend against using the terminology of “utilization distribution” for MKDE and other interpolative methods, and to restrict the use of that phrase to extrapolated distributions [33]. Accordingly, in the zebra case study, the “home range” area was much smaller when estimated with MKDE than with other methods (Table 1, Fig. 1). The isopleth of the MKDE distribution quantifies the process uncertainty around the interpolated path, and the interpolated time budget *during the study*. By contrast, KDEr and AKDEc are designed to delineate asymptotically consistent, statistically robust, conservative buffers around activity centers.

Like KDEr and AKDEc, SE-AKDEc, and E-AKDE extrapolate the utilization probability. But, contrary to KDEr and AKDEc, they include a dose of mechanism (Eq. 1) into the base kernels of the extrapolation (Eq. 5). This yields what can be termed a semi-parametric extrapolation. Compared to AKDEc, the dose of mechanism modifies what the method considers a plausible realization of the movement process. The objective is to combine the asymptotic consistence of AKDE with the biological realism of fully parametric methodologies. One of the main criticism of fully parametric extrapolations [40] is indeed their sensitivity to the goodness of fit of the underlying mechanistic movement model, which the semi-parametric approach partly relaxes. In terms of biological inference, SE-AKDEc, and E-AKDE extrapolate the *potentially accessible resources* under a known set of movement rules and under the constraint that the movement paths must all pass through the recorded locations. Comparing extrapolations from different models can help infer new movement mechanisms or assess model parsimony. Finally, compared to the IBS approach, SE-AKDEc and E-AKDE provide three key advantages: no tuning parameters, full conditioning on the recorded locations, and asymptotic consistency. As highlighted above, a way to better articulate the IBS approach with E-AKDE would be to use IBS to generate the pilot estimate upon which to base the reference function.

## Conclusion

The key message is that it should soon be possible to make the statistically robust, asymptotically consistent alternatives to KDE less bandwidth-limited than they currently are, and make them yield more realistic, less ovoid home range shapes. I introduced new semi-parametric methodologies, based on the step-selection framework [23]. I outlined several avenues for future improvement. I expect E-AKDE to function as part of an iterative process by which semi-mechanistic extrapolations are compared to realized resource use until no significant improvement can be made by adding new movement rules in the extrapolation process. The point would be to give less importance to the time spent at a given location, and more importance to the ratio between availability and use.

## Additional files

Additional file 1: Appendix A: Description of the E-AKDE bandwidth optimizer. (PDF 271 kb)

Additional file 2: Appendix B: Additional method elements: The space-time point process likelihood and Approximate routine to determine whether a linear feature is crossed. (PDF 241 kb)

## Abbreviation

AKDE: autocorrelated kernel density estimator
E-AKDE: autocorrelated kernel density estimator with environmental interactions.
KDE: kernel density estimator
MKDE: movement-based kernel density estimator
