# Supplementary material for: Modified home range kernel density estimators that take environmental interactions into account
Source: Mov Ecol. 2019 May 21;7:16. doi: 10.1186/s40462-019-0161-9 (PMC6530033; doi:10.1186/s40462-019-0161-9)
Supplement: Supplementary file 1 — Appendix A: Description of the E-AKDE bandwidth optimizer. (PDF 271 kb) [file 40462_2019_161_MOESM1_ESM.pdf]

Appendix to "*Modified kernel density estimators that take environmental interactions into account*" by Guillaume Péron

Appendix A

E-AKDE bandwidth optimizer

The elements below serve two purposes: first, making the proof of concept of AKDE [1] more accessible to a wider audience; second, describing how environmental interactions were incorporated into the AKDE bandwidth optimizer.

Throughout, the multivariate Gaussian density function of mean $\boldsymbol{\mu}$ and variance-covariance matrix $\boldsymbol{\Sigma}$ is denoted

$$\varphi(\boldsymbol{r},\boldsymbol{\mu},\boldsymbol{\Sigma}) = \frac{\exp(-{}^{1}/_{2}\,(\boldsymbol{r}-\boldsymbol{\mu})^{\mathrm{T}}\boldsymbol{\Sigma}^{-1}(\boldsymbol{r}-\boldsymbol{\mu})}{\sqrt{det(2\pi\boldsymbol{\Sigma})}}$$

with **r** a vector of dimension *d*=2.

A) Lemma A: "Completing the square"

The product of two Gaussian densities of **r** can be expressed as a product of a Gaussian density independent of **r** and a Gaussian density of **r**.

$$\varphi(\boldsymbol{r},\boldsymbol{\mu_1},\boldsymbol{\Sigma_1})\varphi(\boldsymbol{r},\boldsymbol{\mu_2},\boldsymbol{\Sigma_2}) = \varphi(\boldsymbol{\mu_1},\boldsymbol{\mu_2},\boldsymbol{\Sigma_1}+\boldsymbol{\Sigma_2})\varphi(\boldsymbol{r},\boldsymbol{\mu_C},\boldsymbol{\Sigma_C})$$

where

$$\boldsymbol{\mu_C} = \left(\boldsymbol{\Sigma_1}^{-1}+\boldsymbol{\Sigma_2}^{-1}\right)^{-1}\left(\boldsymbol{\Sigma_1}^{-1}\boldsymbol{\mu_1}+\boldsymbol{\Sigma_2}^{-1}\boldsymbol{\mu_2}\right)$$

$$\boldsymbol{\Sigma_C} = \left(\boldsymbol{\Sigma_1}^{-1}+\boldsymbol{\Sigma_2}^{-1}\right)^{-1}$$

A summarized proof is found in Pedersen et al. (2008 pp.40-41).

This lemma is key to E-AKDE derivation, because the first term in the right-hand product does not depend on ***r*** so can be extracted from spatial integrals. The expression "completing the square" may not be familiar to non-anglophone readers so it seemed worthwhile to explain it in full.

B) Lemma B: Multivariate Gauss Hermite Quadrature

In one dimension, the Gauss Hermite quadrature refers to the use of Hermite polynomials to approximate an integral of the form $\int_{x=-\infty}^{+\infty} f(x)\exp(-x^2/2)\,dx$ with a finite sum of Q terms

$$\int_{x=-\infty}^{+\infty} f(x)\exp(-x^2/2)\,dx \approx \sum_{u=1}^{Q} w_u f(z_u)$$

The weights $\{w_u\}$ and nodes $\{z_u\}$ have been tabularized for various Q values [3]. It is generally recommended to use Q ≥ 15. This quadrature reputedly remains precise even in the presence of a moderate number of discontinuities in *f*.

Now in multiple dimensions, the problem is to compute integrals of the form

$$\int_{\boldsymbol{x}\in\mathbb{R}^d} f(\boldsymbol{x})\frac{\exp\left(-{}^{1}/_{2}\,\boldsymbol{x}^T\boldsymbol{\Sigma}^{-1}\boldsymbol{x}\right)}{\sqrt{\det(2\pi\boldsymbol{\Sigma})}}d\boldsymbol{x}$$

In other words, we want to generalize the 1D Gauss-Hermite quadrature to 2D (or 3D), in order to approximate the above multivariate integral with a finite sum. The problem is therefore to 1) place the nodes in the *d*-dimensional space

in an optimal way relative to the covariance terms in $\boldsymbol{\Sigma}$; and 2) minimize the number of terms in the sum (for a fixed Q) in order to decrease the computing time.

Following Jäkel (2005), the first objective is reached by rotating the summation grid using the spectral decomposition of $\boldsymbol{\Sigma}$, that is the matrix **S** and diagonal matrix $\boldsymbol{\Lambda}$ that yield

$$\boldsymbol{\Sigma} = \mathbf{S}^{\mathrm{T}}\boldsymbol{\Lambda}\mathbf{S}$$

We define $\boldsymbol{\mathcal{A}}(\boldsymbol{\Sigma}) = \mathbf{S}\sqrt{\boldsymbol{\Lambda}}$, we introduce the rotation operator $\boldsymbol{\mathcal{R}}_{d-1}$ that operates d-1 45° rotations in each of the d-1 planes that constitute the d-dimensional environment, and we obtain the preferred set of nodes $\mathbf{z}' = \boldsymbol{\mathcal{A}}(\boldsymbol{\Sigma}) \cdot \boldsymbol{\mathcal{R}}_{d-1} \cdot \mathbf{z}$. This routine is illustrated by Jäkel (2005, in his Fig. 5).

The second objective is reached by "pruning", i.e., removing the nodes with very small contributions to the sum. Following Jäkel (2005), we introduce a cutoff value below which the contribution of the focal node is considered negligible.

The 2D Gauss-Hermite quadrature is then written:

$$\int_{x\in\mathbb{R}^2} f(\boldsymbol{x}) \frac{\exp(-\,^1\!/_2\, \boldsymbol{x}^T\boldsymbol{\Sigma}^{-1}\boldsymbol{x})}{\sqrt{\det(2\pi\boldsymbol{\Sigma})}} d\boldsymbol{x} = \sum_{u=1}^{Q}\sum_{v=1}^{Q} I_{u,v} w_u w_v f\left(\boldsymbol{\mathcal{A}}(\boldsymbol{\Sigma}) \cdot \boldsymbol{\mathcal{R}}_{\mathbf{1}} \cdot \begin{bmatrix} z_u \\ z_v \end{bmatrix}\right)$$

where

$$I_{u,v} = \begin{cases} 1 & \text{if } w_u w_v > \dfrac{w_1 w_{\frac{Q+1}{2}}}{Q} \\ 0 & \text{otherwise} \end{cases}$$

C) Computing and minimizing the Mean Integrated Square Error of E-AKDE

We denote $p(\boldsymbol{r})$ the probability that location ***r*** is utilized by the focal animal. As per the kernel density estimation paradigm, we want to approximate $p(\boldsymbol{r})$ with a sum of *n* unimodal density functions, where *n* is the number of data points. In E-AKDE, each kernel is a weighed multivariate Gaussian distribution:

$$\hat{p}(\boldsymbol{r}) = \frac{1}{n}\sum_{i=1}^{n} K_i^{B^{-1}} W(\boldsymbol{r}|\boldsymbol{R}_i)\varphi(\boldsymbol{r}, \boldsymbol{r_i}, \boldsymbol{\sigma_B})$$

where the scaling constant $K_i^B = \int_{\mathbb{R}^2} W(\boldsymbol{r}|\boldsymbol{R}_i)\varphi(\boldsymbol{r}, \boldsymbol{r_i}, \boldsymbol{\sigma_B})d\boldsymbol{r}$ and other notation as in the main text.

The mean integrated square error (MISE) quantifies the difference between the true distribution and its estimator. Following Fleming et al. (2015) we decompose the MISE into three components

$$MISE(\sigma_B) = \langle \int_\Omega |p(\boldsymbol{r}) - \hat{p}(\boldsymbol{r})|^2 \, d\boldsymbol{r} \rangle$$

$$= \overbrace{\langle \int p(\boldsymbol{r})p(\boldsymbol{r}) \, d\boldsymbol{r} \rangle}^{(1)} - 2\overbrace{\langle \int p(\boldsymbol{r})\hat{p}(\boldsymbol{r}) \, d\boldsymbol{r} \rangle}^{(2)} + \overbrace{\langle \int \hat{p}(\boldsymbol{r})\hat{p}(\boldsymbol{r}) d\boldsymbol{r} \rangle}^{(3)}$$

Following Silverman [5] and most later authors (e.g., [1,6]), we then replace $p(\boldsymbol{r})$ with a mathematically tractable function, a.k.a. reference function, in order to be able to evaluate the MISE (see main text for an important discussion of this). For KDE and AKDE, the reference function is a simple multivariate normal distribution. For E-AKDE, we used a weighed multivariate Gaussian distribution as reference function that we averaged across recorded locations. This yields:

$$p(\boldsymbol{r}) \approx p_{REF}(\boldsymbol{r}) = \frac{1}{n}\sum_{i=1}^{n} K_i^{0^{-1}} W(\boldsymbol{r}|\boldsymbol{R}_i)\varphi(\boldsymbol{r}, \boldsymbol{\mu_0}, \boldsymbol{\sigma_0})$$

where $\boldsymbol{\mu_0} = \langle \boldsymbol{r} \rangle$ and $\boldsymbol{\sigma_0} = \langle (\boldsymbol{r} - \boldsymbol{\mu_0})^T (\boldsymbol{r} - \boldsymbol{\mu_0}) \rangle$, $K_i^0 = \int_{\mathbb{R}^2} W(\boldsymbol{r}|\boldsymbol{R}_i)\varphi(\boldsymbol{r}, \boldsymbol{\mu_0}, \boldsymbol{\sigma_0}) d\boldsymbol{r}$, and other notation as in the main text.

After replacing $p(\boldsymbol{r})$ with $p_{REF}(\boldsymbol{r})$ in the MISE formula, we integrate each of the three terms separately. We first integrate over space (variable ***r***), second over realizations of the movement process (variables $\boldsymbol{r}_i$ and $\boldsymbol{r}_j$), using the lemmas A and B. In doing so we treat the scaling constants *K* as constants, not random variables.

The following is written for 2D applications and using the Ornstein-Uhlenbeck position process with constant mean to model availability (see main text). The framework can accommodate other availability models and 3D situations, but this would require redoing to math below.

First term

$$(1) = \frac{1}{n^2} \sum_{i,j=1}^{n} \langle \int \; K_i^{0^{-1}} W(\boldsymbol{r}|\boldsymbol{R}_i)\varphi(\boldsymbol{r}, \boldsymbol{\mu_0}, \boldsymbol{\sigma_0}) K_j^{0^{-1}} W(\boldsymbol{r}|\boldsymbol{R}_j)\varphi(\boldsymbol{r}, \boldsymbol{\mu_0}, \boldsymbol{\sigma_0}) \; d\boldsymbol{r} \rangle$$

From lemma A

$$K_i^{0^{-1}} W(\boldsymbol{r}|\boldsymbol{R}_i)\varphi(\boldsymbol{r}, \boldsymbol{\mu_0}, \boldsymbol{\sigma_0}) K_j^{0^{-1}} W(\boldsymbol{r}|\boldsymbol{R}_j)\varphi(\boldsymbol{r}, \boldsymbol{\mu_0}, \boldsymbol{\sigma_0}) = K_i^{0^{-1}} K_j^{0^{-1}} W(\boldsymbol{r}|\boldsymbol{R}_i) W(\boldsymbol{r}|\boldsymbol{R}_j) \; \varphi(\boldsymbol{\mu_0}, \boldsymbol{\mu_0}, \boldsymbol{2\sigma_0})\varphi(\boldsymbol{r}, \boldsymbol{\mu_0}, \boldsymbol{2\sigma_0})$$

Integration over space

From lemma B

$$\int \; K_i^{0^{-1}} K_j^{0^{-1}} W(\boldsymbol{r}|\boldsymbol{R}_i) W(\boldsymbol{r}|\boldsymbol{R}_j) \; \varphi(\boldsymbol{\mu_0}, \boldsymbol{\mu_0}, \boldsymbol{2\sigma_0})\varphi(\boldsymbol{r}, \boldsymbol{\mu_0}, \boldsymbol{2\sigma_0}) \; d\boldsymbol{r}$$
$$= K_i^{0^{-1}} K_j^{0^{-1}} \sum_{u,v=1}^{Q} I_{u,v} w_u w_v \widetilde{W}_i(z_u, z_v, \boldsymbol{\mu_0}, \boldsymbol{2\sigma_0}) \widetilde{W}_j(z_u, z_v, \boldsymbol{\mu_0}, \boldsymbol{2\sigma_0})$$

with $\widetilde{W}_i(z_u, z_v, \boldsymbol{\mu}, \boldsymbol{\sigma}) = W\left(\boldsymbol{\mathcal{A}}(\boldsymbol{\sigma}) \cdot \boldsymbol{\mathcal{R}_1} \cdot \begin{bmatrix} z_u \\ z_v \end{bmatrix} + \boldsymbol{\mu} \middle| \boldsymbol{R}_i\right)$.

Note that this term does not depend on the bandwidth so in effect does not need to be computed.

Second term

$$(2) = \frac{1}{n^2} \sum_{i,j=1}^{n} \langle \int \; K_i^{0^{-1}} W(\boldsymbol{r}|\boldsymbol{R}_i)\varphi(\boldsymbol{r}, \boldsymbol{\mu_0}, \boldsymbol{\sigma_0}) K_j^{B^{-1}} W(\boldsymbol{r}|\boldsymbol{R}_j)\varphi(\boldsymbol{r}, \boldsymbol{r_j}, \boldsymbol{\sigma_B}) \; d\boldsymbol{r} \rangle$$

From lemma A

$$K_i^{0^{-1}} W(\boldsymbol{r}|\boldsymbol{R}_i)\varphi(\boldsymbol{r}, \boldsymbol{\mu_0}, \boldsymbol{\sigma_0}) K_j^{B^{-1}} W(\boldsymbol{r}|\boldsymbol{R}_j)\varphi(\boldsymbol{r}, \boldsymbol{r_j}, \boldsymbol{\sigma_B})$$
$$= K_i^{0^{-1}} K_j^{B^{-1}} W(\boldsymbol{r}|\boldsymbol{R}_i) W(\boldsymbol{r}|\boldsymbol{R}_j) \; \varphi(\boldsymbol{\mu_0}, \boldsymbol{r_j}, \boldsymbol{\sigma_0} + \boldsymbol{\sigma_B})\varphi(\boldsymbol{r}, \boldsymbol{m}_1(\boldsymbol{r_j}), \boldsymbol{\sigma}_1)$$

With $\boldsymbol{\sigma}_1 = (\boldsymbol{\sigma}_0{}^{-1} + \boldsymbol{\sigma}_B{}^{-1})^{-1}$ and $\boldsymbol{m}_1(\boldsymbol{r_j}) = \boldsymbol{\sigma}_1 \cdot (\boldsymbol{\sigma_0}^{-1}\boldsymbol{\mu_0} + \boldsymbol{\sigma_B}^{-1}\boldsymbol{r_j})$

Integration over space

From lemma B

$$\int K_i^{0^{-1}} K_j^{B^{-1}} W(\boldsymbol{r}|\boldsymbol{R}_i) W(\boldsymbol{r}|\boldsymbol{R}_j) \; \varphi(\boldsymbol{\mu_0}, \boldsymbol{r_j}, \boldsymbol{\sigma_0} + \boldsymbol{\sigma_B})\varphi(\boldsymbol{r}, \boldsymbol{m_1}(\boldsymbol{r_j}), \boldsymbol{\sigma}_1) \; d\boldsymbol{r}$$
$$= K_i^{0^{-1}} K_j^{B^{-1}} \sum_{u,v=1}^{Q} I_{u,v} w_u w_v \; \varphi(\boldsymbol{\mu_0}, \boldsymbol{r_j}, \boldsymbol{\sigma_0} + \boldsymbol{\sigma_B}) \widetilde{W}_i(z_u, z_v, \boldsymbol{m}_1(\boldsymbol{r_j}), \boldsymbol{\sigma}_1) \widetilde{W}_j(z_u, z_v, \boldsymbol{m}_1(\boldsymbol{r_j}), \boldsymbol{\sigma}_1)$$

Integration across realizations

By definition

$$\langle\, \varphi(\boldsymbol{\mu_0}, \boldsymbol{r_j}, \boldsymbol{\sigma_0} + \boldsymbol{\sigma_B})\widetilde{W}_i\big(z_u, z_v, \boldsymbol{m}_1(\boldsymbol{r_j}), \boldsymbol{\sigma}_1\big)\widetilde{W}_j\big(z_u, z_v, \boldsymbol{m}_1(\boldsymbol{r_j}), \boldsymbol{\sigma}_1\big)\,\rangle$$
$$= \int \varphi(\boldsymbol{\mu_0}, \boldsymbol{r_j}, \boldsymbol{\sigma_0} + \boldsymbol{\sigma_B})\widetilde{W}_i\big(z_u, z_v, \boldsymbol{m}_1(\boldsymbol{r_j}), \boldsymbol{\sigma}_1\big)\widetilde{W}_j\big(z_u, z_v, \boldsymbol{m}_1(\boldsymbol{r_j}), \boldsymbol{\sigma}_1\big)K_j^{-1}W\big(\boldsymbol{r_j}\big|\boldsymbol{R_j}\big)\varphi\big(\boldsymbol{r_j}, \boldsymbol{\mu_j}, \boldsymbol{\sigma_0}\big)\, d\boldsymbol{r_j}$$

with $\boldsymbol{\mu_j} = \boldsymbol{\mu_0} + \big(\boldsymbol{r_j} - \boldsymbol{\mu_0}\big)\cdot\left[1 - e^{\frac{(t_i - t_{i-1})}{\tau}}\right]$ and $K_j = \int_\Omega W(\boldsymbol{r}|\boldsymbol{R}_j)\varphi\big(\boldsymbol{r}, \boldsymbol{\mu_j}, \boldsymbol{\sigma_0}\big)d\boldsymbol{r}$

Therefore from lemma B

$$\langle\, \varphi(\boldsymbol{\mu_0}, \boldsymbol{r_j}, \boldsymbol{\sigma_0} + \boldsymbol{\sigma_B})\widetilde{W}_i\big(z_u, z_v, \boldsymbol{m}_1(\boldsymbol{r_j}), \boldsymbol{\sigma}_1\big)\widetilde{W}_j\big(z_u, z_v, \boldsymbol{m}_1(\boldsymbol{r_j}), \boldsymbol{\sigma}_1\big)\,\rangle$$
$$= \sum_{u',v'=1}^{Q} I_{u',v'}w_{u'}w_{v'}f_{ijuv}\left(\boldsymbol{\mathcal{A}}(\boldsymbol{\sigma_0})\cdot\boldsymbol{\mathcal{R}_1}\cdot\begin{bmatrix} z_u \\ z_v \end{bmatrix} + \boldsymbol{\mu_j}\right)$$

where $f_{ijuv}(\boldsymbol{r}) = \varphi(\boldsymbol{\mu_0}, \boldsymbol{r}, \boldsymbol{\sigma_0} + \boldsymbol{\sigma_B})\widetilde{W}_i(z_u, z_v, \boldsymbol{m}_1(\boldsymbol{r}), \boldsymbol{\sigma}_1)\widetilde{W}_j(z_u, z_v, \boldsymbol{m}_1(\boldsymbol{r}), \boldsymbol{\sigma}_1)K_j^{-1}W\big(\boldsymbol{r}\big|\boldsymbol{R}_j\big)$

Third term

$$(3) = \frac{1}{n^2}\sum_{i,j=1}^{n}\langle\int K_i^{B^{-1}}W(\boldsymbol{r}|\boldsymbol{R}_i)\varphi(\boldsymbol{r}, \boldsymbol{r_i}, \boldsymbol{\sigma_B})K_j^{B^{-1}}W\big(\boldsymbol{r}\big|\boldsymbol{R}_j\big)\varphi\big(\boldsymbol{r}, \boldsymbol{r_j}, \boldsymbol{\sigma_B}\big)\, d\boldsymbol{r}\rangle$$

From lemma A

$$K_i^{B^{-1}}W(\boldsymbol{r}|\boldsymbol{R}_i)\varphi(\boldsymbol{r}, \boldsymbol{r_i}, \boldsymbol{\sigma_B})K_j^{B^{-1}}W\big(\boldsymbol{r}\big|\boldsymbol{R}_j\big)\varphi\big(\boldsymbol{r}, \boldsymbol{r_j}, \boldsymbol{\sigma_B}\big)$$
$$= K_i^{B^{-1}}K_j^{B^{-1}}W(\boldsymbol{r}|\boldsymbol{R}_i)W\big(\boldsymbol{r}\big|\boldsymbol{R}_j\big)\,\varphi\big(\boldsymbol{r_i}, \boldsymbol{r_j}, 2\boldsymbol{\sigma_B}\big)\varphi\left(\boldsymbol{r}, \frac{\boldsymbol{r_i} + \boldsymbol{r_j}}{2}, \frac{\boldsymbol{\sigma_B}}{2}\right)$$

Integration over space

From lemma B

$$\int K_i^{B^{-1}}K_j^{B^{-1}}W(\boldsymbol{r}|\boldsymbol{R}_i)W\big(\boldsymbol{r}\big|\boldsymbol{R}_j\big)\,\varphi\big(\boldsymbol{r_i}, \boldsymbol{r_j}, 2\boldsymbol{\sigma_B}\big)\varphi\left(\boldsymbol{r}, \frac{\boldsymbol{r_i} + \boldsymbol{r_j}}{2}, \frac{\boldsymbol{\sigma_B}}{2}\right)\, d\boldsymbol{r}$$
$$= K_i^{B^{-1}}K_j^{B^{-1}}\sum_{u,v=1}^{Q} I_{u,v}w_u w_v\,\varphi\big(\boldsymbol{r_i}, \boldsymbol{r_j}, 2\boldsymbol{\sigma_B}\big)\widetilde{W}_i\left(z_u, z_v, \frac{\boldsymbol{r_i} + \boldsymbol{r_j}}{2}, \frac{\boldsymbol{\sigma_B}}{2}\right)\widetilde{W}_j\left(z_u, z_v, \frac{\boldsymbol{r_i} + \boldsymbol{r_j}}{2}, \frac{\boldsymbol{\sigma_B}}{2}\right)$$

Integration across realizations

By definition

$$\langle\varphi\big(\boldsymbol{r_i}, \boldsymbol{r_j}, 2\boldsymbol{\sigma_B}\big)\widetilde{W}_i\left(z_u, z_v, \frac{\boldsymbol{r_i} + \boldsymbol{r_j}}{2}, \frac{1}{2}\boldsymbol{\sigma_B}\right)\widetilde{W}_j\left(z_u, z_v, \frac{\boldsymbol{r_i} + \boldsymbol{r_j}}{2}, \frac{1}{2}\boldsymbol{\sigma_B}\right)\rangle$$
$$= \iint \varphi\big(\boldsymbol{r_i}, \boldsymbol{r_j}, 2\boldsymbol{\sigma_B}\big)\widetilde{W}_i\left(z_u, z_v, \frac{\boldsymbol{r_i} + \boldsymbol{r_j}}{\boldsymbol{2}}, \frac{1}{2}\boldsymbol{\sigma_B}\right)\widetilde{W}_j\left(z_u, z_v, \frac{\boldsymbol{r_i} + \boldsymbol{r_j}}{\boldsymbol{2}}, \frac{1}{2}\boldsymbol{\sigma_B}\right)\, p\begin{pmatrix} \boldsymbol{r_i} \\ \boldsymbol{r_j} \end{pmatrix}\, d\boldsymbol{r_i}d\boldsymbol{r_j}$$

where the joint distribution of $\boldsymbol{r}_i$ and $\boldsymbol{r}_j$ is $p\begin{pmatrix} \boldsymbol{r_i} \\ \boldsymbol{r_j} \end{pmatrix} = K_{ij}{}^{-1}W(\boldsymbol{r_i}|\boldsymbol{R}_i)W\big(\boldsymbol{r_j}\big|\boldsymbol{R}_j\big)\varphi\left(\begin{pmatrix} \boldsymbol{r_i} \\ \boldsymbol{r_j} \end{pmatrix}, \begin{pmatrix} \boldsymbol{\mu_i} \\ \boldsymbol{\mu_j} \end{pmatrix}, \begin{pmatrix} \boldsymbol{\sigma_0} & \boldsymbol{\sigma_{ij}} \\ \boldsymbol{\sigma_{ij}} & \boldsymbol{\sigma_0} \end{pmatrix}\right)$, with $\boldsymbol{\sigma_{ij}} = \boldsymbol{\sigma_0}\cdot\left[1 - e^{-2|t_j - t_i|/\tau}\right]$, $K_{ij} = \int_{\mathbb{R}^2}\int_{\mathbb{R}^2} W(\boldsymbol{r_i}|\boldsymbol{R}_i)W\big(\boldsymbol{r_j}\big|\boldsymbol{R}_j\big)\varphi\left(\begin{pmatrix} \boldsymbol{r_i} \\ \boldsymbol{r_j} \end{pmatrix}, \begin{pmatrix} \boldsymbol{\mu_i} \\ \boldsymbol{\mu_j} \end{pmatrix}, \begin{pmatrix} \boldsymbol{\sigma_0} & \boldsymbol{\sigma_{ij}} \\ \boldsymbol{\sigma_{ij}} & \boldsymbol{\sigma_0} \end{pmatrix}\right)d\boldsymbol{r_i}d\boldsymbol{r_j}$, and other notation as above.

Therefore from lemma B

$$\langle\varphi(\boldsymbol{r_i},\boldsymbol{r_j},2\boldsymbol{\sigma_B})\widetilde{W}_i\left(z_u,z_v,\frac{\boldsymbol{r_i}+\boldsymbol{r_j}}{2},\frac{1}{2}\boldsymbol{\sigma_B}\right)\widetilde{W}_j\left(z_u,z_v,\frac{\boldsymbol{r_i}+\boldsymbol{r_j}}{2},\frac{1}{2}\boldsymbol{\sigma_B}\right)\rangle$$

$$= \sum_{u',v',u'',v''=\mathbf{1}}^{Q} I_{u',v'}I_{u'',v''}w_{u'}w_{v'}w_{u''}w_{v''}g_{ijuv}\left(\boldsymbol{\mathcal{A}}\left(\begin{pmatrix}\boldsymbol{\sigma_0} & \boldsymbol{\sigma_{ij}}\\ \boldsymbol{\sigma_{ij}} & \boldsymbol{\sigma_0}\end{pmatrix}\right)\cdot\boldsymbol{\mathcal{R}_3}\cdot\begin{bmatrix}z_{u'}\\ z_{v'}\\ z_{u''}\\ z_{v''}\end{bmatrix}+\begin{pmatrix}\boldsymbol{\mu_i}\\ \boldsymbol{\mu_j}\end{pmatrix}\right)$$

with $g_{ijuv}\left(\begin{pmatrix}\boldsymbol{r}\\ \boldsymbol{s}\end{pmatrix}\right) = K_{ij}{}^{-1}\varphi(\boldsymbol{r},\boldsymbol{s},2\boldsymbol{\sigma_B})\widetilde{W}_i\left(z_u,z_v,\frac{r+s}{2},\frac{1}{2}\boldsymbol{\sigma_B}\right)\widetilde{W}_j\left(z_u,z_v,\frac{r+s}{2},\frac{1}{2}\boldsymbol{\sigma_B}\right)W(\boldsymbol{r}|\boldsymbol{R}_i)W(\boldsymbol{s}|\boldsymbol{R}_j)$

References cited in this appendix

1. Fleming CH, Fagan WF, Mueller T, Olson KA, Leimgruber P, Calabrese JM. Rigorous home-range estimation with movement data: A new autocorrelated kernel-density estimator. Ecology [Internet]. 2015 [cited 2015 Apr 9];96:1182–8. Available from: http://dx.doi.org/10.1890/14-2010.1%5Cnhttp://www.esajournals.org/doi/pdf/10.1890/14-2010.1

2. Pedersen MS, Baxter B, Templeton B, Rishøj C, Theobald DL, Hoegh-rasmussen E, et al. The Matrix Cookbook [Internet]. 2008. Available from: http://matrixcookbook.com

3. Golub GH, Welsch JH. Calculation of Gauss quadrature rules. Math. Comput. [Internet]. 1969 [cited 2016 Aug 31];23:221–221. Available from: http://www.ams.org/jourcgi/jour-getitem?pii=S0025-5718-69-99647-1

4. Jäkel P. A note on multivariate Gauss-Hermite quadrature. London; 2005.

5. Silverman BW. Density estimation for statistics and data analysis. London: Chapman and Hall; 1986.

6. Kie JG. A rule-based ad hoc method for selecting a bandwidth in kernel home-range analyses. Anim. Biotelemetry [Internet]. 2013 [cited 2016 Aug 30];1:13. Available from: http://animalbiotelemetry.biomedcentral.com/articles/10.1186/2050-3385-1-13
