# Supplementary material for: Modified home range kernel density estimators that take environmental interactions into account
Source: Mov Ecol. 2019 May 21;7:16. doi: 10.1186/s40462-019-0161-9 (PMC6530033; doi:10.1186/s40462-019-0161-9)

Appendix to “*Modified kernel density estimators that take environmental interactions into account*” by Guillaume Péron

Appendix B

Additional method elements (parts A and B)

A) The space-time point process likelihood

As explained by Johnson et al. (2013), I used the Berman and Turner (1992) quadrature of the space-time point process likelihood.

$$L(\boldsymbol{\theta}) = \sum_{j,k} q_{jk} \cdot \left[z_{jk} \cdot \log \lambda\left(\boldsymbol{r}_{jk}, t_j \middle| \boldsymbol{R}_{t_j-1}\right) - \lambda\left(\boldsymbol{r}_{jk}, t_j \middle| \boldsymbol{R}_{t_j-1}\right)\right]$$

where λ is called the conditional intensity function, $\{t_j; j = 1 \dots J\}$ contains the timestamps of the tracking data, augmented with a large number of temporal quadrature points, $\{\boldsymbol{r}_{jk}; j = 1 \dots J, k = 1 \dots K\}$ contains the observed locations augmented with a large number of spatial quadrature points, q_{jk} is the volume surrounding quadrature point $(\boldsymbol{r}_{jk}, t_j)$, $z_{jk} = 1/q_{jk}$ if $(\boldsymbol{r}_{jk}, t_j)$ corresponds to an observation, 0 otherwise, and $\boldsymbol{\theta}$ denotes the parameters to be estimated.

In our case, the conditional intensity function was $\lambda(\boldsymbol{r}, t|\boldsymbol{R}_{t-1}) = W(\boldsymbol{r}|\boldsymbol{R}_{t-1}, t)g_a(\boldsymbol{r}|\boldsymbol{R}_{t-1})$ (notations as in the main text).

To fully benefit from the computing time boost made possible by the Berman and Turner quadrature, I approximated the availability function g_a by a Brownian availability window with parameter $\tilde{\sigma}$ to be estimated. Thereby, λ became a log-linear function of $\boldsymbol{\theta}$, and $L(\boldsymbol{\theta})$ became the likelihood of a Poisson generalized linear model with observations z_{jk} and lights q_{jk}. I could use R (or any statistical software) to estimate the parameters.

I used a resolution of 90 meters and 30 minutes for the Berman and Turner quadrature and assumed a maximum speed of 0.7 m/s to define the sampling window.

B) Technical tricks

The computational complexity of the E-AKDE bandwidth optimizer is in $O(Q^6 NlogN)$ with a large multiplication factor (Appendix A), meaning that the computation would take months on a desktop computer. The multiplication factor could be made smaller by removing the barrier crossing feature, but the computing time would still be considerable. Most of the time is spent in step 2. I used a few techniques and approximations to tentatively reduce the computing time of step 2 to a tractable value. In spite of these, the computing time is still too large for widespread application, hence the suggestion of a simplified approach, denoted SE-AKDEc, at the end of this section.

1) Scaling constants

The algorithm requires the computation of scaling constants for each recorded location $\boldsymbol{r}_{i,}$ so that the weighed multivariate Gaussian distributions sum to one (Appendix A). I simplified the computation by noting that the recorded locations came in three clusters in my application case (see next section). So, I computed one scaling constant per cluster instead of one per location. I computed the scaling constants with a numerical quadrature (e.g., Johnson et al. 2008b), by sampling 500 points from the uniform distribution, and 500 points from the bivariate normal distribution with mean the centroid of the cluster and variance the variance of the OU-p movement process as estimated at step 1. The source code is in Appendix D.

2) Barrier crossing

Preliminary analyses indicated that the most time-consuming part of the algorithm was determining whether a linear feature (barrier) was crossed between each pair of points considered in the algorithm. I devised an approximate routine that reduced the number of operations required to determine whether a linear feature was crossed (Appendix B), meaning that adding the border crossing feature to the model was not very costly anymore. The source code is in Appendix C or D.

3) Gauss-Hermite quadrature

Other than the scaling constants, I computed the spatial integrals in step 2 using the Gauss-Hermite quadrature (Appendix A), thereby obtaining an algorithm in $O(Q^6 NlogN)$ instead of $O(N^7 logN)$. The source code is in Appendix D.

4) By-passing step 2 altogether

Given how time-consuming step 2 is, I considered a simplified alternative in which I only accounted for environmental interactions at the final step (step 3). In this simplified version, I ignored environmental interactions when I estimated the bandwidth, i.e., I applied Fleming et al. (2015) routine to estimate the bandwidth. In this case, I corrected for the reference function approximation bias after step 3. I did not account for environmental interactions when I computed the amount of bias to be removed. I denote the whole procedure SE-AKDEc (simplified autocorrelated kernel density estimator with environmental interaction and bias correction). In SE-AKDEc, I computed all the scaling constants (not one constant per cluster) because the need to reduce computing time was less acute than in E-AKDE. The source code is in Appendix C.

C) Approximate routine to determine whether a linear feature is crossed

The linear feature is represented by the thick black jagged line. I compute a spatially explicit variable v with value 0, 1, 2, or 3 depending on the position relative to the linear feature (different shading types). Determining whether the linear feature is crossed when going between two points A and B then becomes a simple raster manipulation in most cases.

If $v_A = 2$ and $v_B = 3$, then the feature is considered crossed when going from A to B or inversely. Conversely, if $v_A = v_B$, $v_A = 1$ and $v_B = 3$, or if $v_A = 0$ and $v_B = 2$, then the feature is considered not crossed. In the remaining cases, for example if $v_A = 1$ and $v_B = 2$, I apply the usual geometric routine to determine whether the line segment [AB] intersects the linear feature.

An important assumption of this approximate routine is that the animal will not feel the resistance of the linear feature on its movement if the detour to avoid crossing the feature is small compared to the length of the movement step.

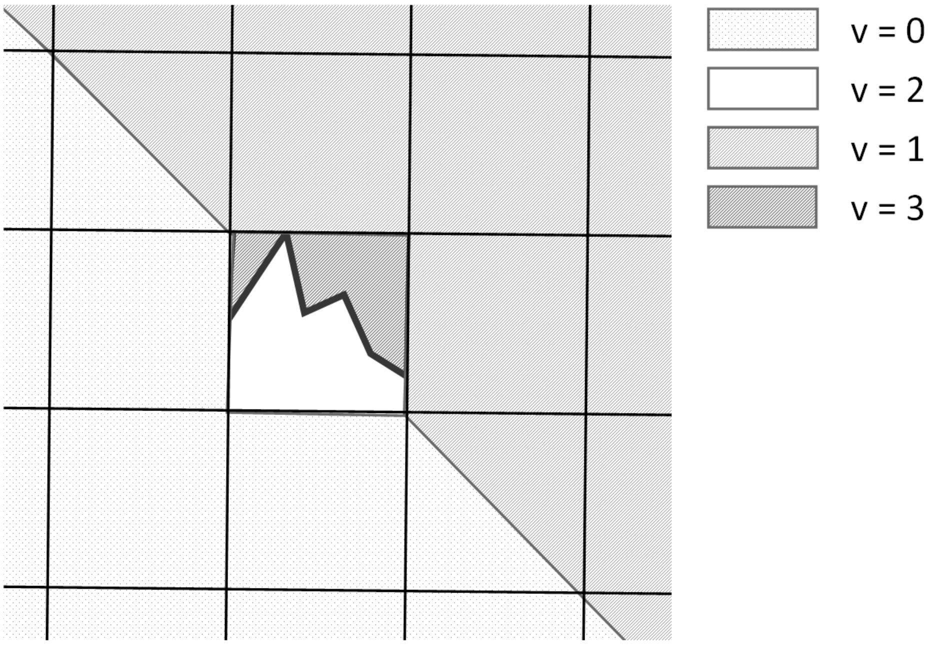

Supplement: Supplementary file 2 — Appendix B: Additional method elements: The space-time point process likelihood and Approximate routine to determine whether a linear feature is crossed. (PDF 241 kb) [file 40462_2019_161_MOESM2_ESM.pdf]
